# Exploring the impact of cultural intelligence on multicultural literacy in university students: a serial mediation model of cultural exposure and cross-cultural communication skills

**DOI:** 10.3389/fpsyg.2025.1661899

**Published:** 2025-09-19

**Authors:** Liu Haikuo

**Affiliations:** Hanyang University, Seoul, Republic of Korea

**Keywords:** cultural intelligence, cultural exposure, cross-cultural communication skills, multicultural literacy, university students, serial mediation, China, structural equation modeling

## Abstract

**Introduction:**

Cultural intelligence plays a central role in shaping students' ability to navigate diverse cultural environments, yet the mechanisms through which it influences multicultural literacy remain underexplored. This study investigates a serial mediation model to examine how cultural intelligence indirectly enhances multicultural literacy through two psychological pathways: cultural exposure and cross-cultural communication skills.

**Methods:**

Data were collected from 412 university students in China using stratified random sampling, with validated instruments measuring each construct. Structural equation modeling using SmartPLS confirmed the reliability and discriminant validity of all constructs.

**Results:**

The results show that cultural intelligence has significant positive effects on both cultural exposure and cross-cultural communication skills. In turn, cultural exposure not only directly enhances multicultural literacy but also facilitates the development of communication skills, which subsequently contributes to multicultural literacy through a sequential mediation pathway.

**Discussion:**

These findings offer theoretical and practical insights into how institutions can foster students' intercultural competence by enhancing both their opportunities for exposure and their interpersonal communication abilities.

## Introduction

Cultural diversity has become a defining feature of modern higher education (Dodd et al., 2022; Zhou and Colomer, 2024), with students increasingly exposed to global perspectives both inside and outside the classroom. In such multicultural contexts, the ability to understand, adapt to, and engage with diverse cultures—often referred to as multicultural literacy—has emerged as a key competence for academic success, personal growth, and global citizenship (Steyn and Vanyoro, 2024). Multicultural literacy encompasses not only the knowledge of cultural norms and values but also the attitudes and skills necessary to interact respectfully and effectively across cultural boundaries (Ahadiyyah et al., 2024; Tien, 2023). As the demand for interculturally competent graduates grows, understanding the antecedents that shape students' multicultural literacy has become an essential focus of educational research.

One psychological factor frequently linked to students' intercultural capacity is cultural intelligence. Defined as an individual's capability to function and manage effectively in culturally diverse settings, cultural intelligence is believed to play a foundational role in how students perceive, process, and respond to intercultural experiences (Mangla, 2021). However, the relationship between cultural intelligence and multicultural literacy is not necessarily direct or linear. Students may possess high levels of cultural intelligence but still lack meaningful opportunities or mechanisms to translate that potential into actual intercultural understanding and literacy. This suggests the presence of intermediary processes that warrant deeper examination.

In conceptualizing the mechanisms through which cultural intelligence contributes to multicultural literacy, cultural exposure emerges as a central experiential mediator. Cultural exposure refers to the extent and frequency of engagement individuals have with culturally diverse experiences, including but not limited to cross-national friendships, participation in intercultural events, study-abroad programs, and consumption of global media (Clark et al., 2022; Sharma and Rakhimova, 2024). From a social learning perspective, such interactions provide the situational input necessary for individuals to activate, test, and refine their cultural knowledge and awareness (Fonagy et al., 2022; Zhou and Schofield, 2024). Exposure to different cultural practices not only stimulates reflective thinking about one's own cultural assumptions but also deepens appreciation of cultural variability, which is foundational to multicultural literacy. Moreover, prior studies suggest that repeated intercultural contact strengthens perspective-taking and adaptive learning, both of which are critical for literacy across cultural domains (Gweon, 2021; Lindquist et al., 2022; Nguyen et al., 2025).

In parallel, cross-cultural communication skills represent a key behavioral competence that may mediate the relationship between cultural intelligence and multicultural literacy. These skills involve the capacity to interpret culturally embedded cues, adjust one's verbal and nonverbal behavior appropriately, and engage in effective interaction with individuals from different backgrounds (Aririguzoh, 2022; Pang et al., 2024). Theoretically grounded in communication competence and intercultural sensitivity models, such skills are not simply mechanical but are shaped by one's cultural mindset and cognitive flexibility—attributes closely linked to cultural intelligence (Kour and Jyoti, 2022; Thu, 2024). Effective cross-cultural communication enables individuals to navigate misunderstandings, build trust, and foster mutual understanding, thereby transforming cultural knowledge into meaningful interpersonal engagement (Chen, 2024; Kai Liao et al., 2021; Xia et al., 2024). In this sense, cross-cultural communication operates as a behavioral conduit through which cognitive and affective dimensions of cultural intelligence are actualized into multicultural literacy.

Despite their conceptual importance, these mediating factors have rarely been integrated into a single explanatory model. Most previous studies have either examined cultural exposure or communication skills in isolation, or have assumed that their influence is secondary to other structural factors, such as curriculum or institutional policy (Ogbogu et al., 2022; Sahadevan and Sumangala, 2021). Furthermore, while there is growing recognition that psychological traits and experiential variables interact in complex ways to shape intercultural competence, few empirical studies have tested these dynamics within the same analytical framework. It is also plausible that these mechanisms may reinforce each other over time; for example, greater cultural exposure can enhance communicative adaptability, while improved cross-cultural communication can further deepen engagement with diverse contexts (Passarelli and Kolb, 2023; Presbitero, 2021). Considering such potential interplay offers a richer perspective on how multicultural literacy develops, especially in relatively homogeneous contexts like Mainland China, where internationalization levels and intercultural opportunities vary across campuses (Qiu et al., 2024; Liu and Lim, 2024). This has resulted in a fragmented understanding of how multicultural literacy develops at the individual level, especially within non-Western educational contexts.

To address this gap, the present study proposes and tests a conceptual model in which cultural intelligence indirectly enhances multicultural literacy through both cultural exposure and cross-cultural communication skills, while recognizing that these developmental processes may interact dynamically. The model is tested among university students in China, where rapid internationalization of higher education has created both opportunities and challenges for intercultural learning (Qiu et al., 2024). By investigating these relationships within a culturally specific yet globally relevant setting, this study contributes to a more integrated understanding of how psychological and experiential factors jointly support the development of multicultural literacy in higher education.

In light of the aforementioned discussion, the following research questions are posited:

RQ1: To what extent does cultural intelligence influence multicultural literacy among university students?
RQ2: Through what experiential and behavioral mechanisms does cultural intelligence exert its influence on multicultural literacy, particularly via cultural exposure and cross-cultural communication skills?

### Relationship between cultural intelligence and multicultural literacy

Prior studies consistently indicate that individuals with higher levels of cultural intelligence are more likely to succeed in multicultural environments and demonstrate a stronger orientation toward inclusive, reflective, and globally informed attitudes. Research has shown that cultural intelligence enhances individuals' ability to engage meaningfully with cultural differences and interpret intercultural situations with greater insight (Skaria and Montayre, 2023; Sousa et al., 2023). Several studies report significant positive correlations between cultural intelligence and outcomes closely related to multicultural literacy, such as intercultural competence, global mindset, and cultural sensitivity (Drame et al., 2021; Shliakhovchuk, 2021). Empirical findings further suggest that cultural intelligence facilitates deeper understanding and appreciation of diverse worldviews, which are central features of multicultural literacy (Abdulahi et al., 2024). In educational contexts, culturally intelligent students have been found to adapt more effectively to diverse classrooms and demonstrate higher levels of perspective-taking and cultural reflection (Bratianu et al., 2021). Moreover, cultural intelligence has been identified as a reliable predictor of students' preparedness for global citizenship and intercultural engagement (Figueroa and Hofhuis, 2024). On the basis of these findings, there is strong empirical support to infer a direct positive relationship between cultural intelligence and multicultural literacy.

H1: Cultural intelligence is positively associated with multicultural literacy.

### Mediating role of cultural exposure

Cultural intelligence has been widely linked with individuals' propensity to engage in intercultural experiences and seek diverse cultural encounters. Individuals with high levels of cultural intelligence are more likely to pursue and create opportunities for cultural learning, such as joining multicultural student organizations, participating in cultural exchange events, or building relationships across ethnic or national boundaries (Kai Liao et al., 2021; Kour and Jyoti, 2022). Empirical studies have shown that cultural intelligence predicts both the quantity and quality of intercultural exposure in academic and professional settings (Baratipour et al., 2021). This tendency may stem from the motivational and behavioral components of cultural intelligence, which drive individuals to explore cultural difference with confidence, openness, and adaptive social behavior (Jurásek and Wawrosz, 2024). Consequently, students with high cultural intelligence are more inclined to immerse themselves in culturally diverse contexts, resulting in richer experiential learning opportunities.

Cultural exposure, in turn, has been identified as a key experiential factor influencing multicultural literacy. Theoretical models rooted in experiential learning suggest that repeated intercultural encounters offer individuals a chance to reflect on differences, question assumptions, and develop a more nuanced understanding of cultural plurality (Passarelli and Kolb, 2023). Students who engage in frequent and meaningful intercultural experiences are more likely to demonstrate awareness of global issues, appreciation of diversity, and sensitivity to power dynamics across cultures—all central components of multicultural literacy (Dodd et al., 2022; Zhou and Colomer, 2024). Previous research indicates that students who have lived abroad, interacted with international peers, or attended culturally themed events tend to score higher on measures of intercultural competence, global citizenship, and inclusive attitudes (Figueroa and Hofhuis, 2024; Wawrosz and Jurásek, 2021). Thus, cultural exposure provides the environmental context in which multicultural literacy is not only developed but also reinforced through real-world application.

Given that cultural intelligence fosters greater engagement with diverse cultural contexts, and that such engagement serves as a catalyst for developing multicultural understanding, cultural exposure is likely to serve as a mediating mechanism in this relationship. This inference is supported by studies showing that the effects of cultural intelligence on various intercultural outcomes are often indirect and depend on actual interaction with culturally dissimilar others (Baratipour et al., 2021). Through exposure, culturally intelligent students activate and refine their cognitive and affective resources in authentic intercultural situations, ultimately enhancing their multicultural literacy. This suggests a process in which cultural intelligence leads students to seek out diverse cultural experiences, which in turn facilitate their literacy in navigating and understanding multicultural environments.

Based on the preceding discussion, the following hypotheses are proposed:

H2a: Cultural intelligence is positively associated with cultural exposure.
H2b: Cultural exposure is positively associated with multicultural literacy.
H2c: Cultural exposure mediates the relationship between cultural intelligence and multicultural literacy.

### Mediating role of cross-cultural communication skills

Individuals with high cultural intelligence tend to demonstrate superior interpersonal adaptability and communicative flexibility in intercultural contexts. As cultural intelligence enhances one's awareness of cultural norms and promotes sensitivity to diverse communicative patterns, it is frequently associated with stronger cross-cultural communication skills (Xia et al., 2024). Culturally intelligent individuals are more capable of decoding subtle verbal and nonverbal cues, adjusting their speech or behavior according to cultural context, and engaging in interactions that are respectful and culturally appropriate (Majda et al., 2021). Prior research indicates that cultural intelligence fosters not only the motivation to engage with individuals from different backgrounds but also the ability to do so effectively, especially in complex or ambiguous situations (Shadiev et al., 2021). Therefore, it is plausible to infer that students with higher levels of cultural intelligence are more likely to develop refined cross-cultural communication skills through their repeated exposure to diverse social and linguistic environments.

Cross-cultural communication skills, in turn, are essential for demonstrating multicultural literacy in practice. These skills allow individuals to go beyond theoretical understanding and actively participate in intercultural dialogue, perspective-taking, and collaborative learning (Hashimoto, 2023). When students possess the ability to communicate clearly, sensitively, and flexibly across cultures, they are more capable of navigating multicultural settings, addressing misunderstandings, and fostering mutual respect. Research shows that students with strong cross-cultural communication competence are more likely to demonstrate openness to cultural diversity, engage in reflective dialogue, and resolve intercultural conflicts constructively—key behaviors aligned with multicultural literacy (Aririguzoh, 2022; Imbrie et al., 2021). Communication competence thus serves as a functional expression of multicultural understanding, translating knowledge into effective action within culturally diverse learning environments.

Building on this logic, cross-cultural communication skills may operate as a behavioral mechanism through which cultural intelligence exerts its influence on multicultural literacy. While cultural intelligence provides the foundational cognitive and motivational resources, communication skills serve as the channel through which these resources are enacted in real-world intercultural interactions. Several studies have suggested that the relationship between cultural intelligence and intercultural effectiveness is often mediated by communication behavior, particularly in student populations navigating diverse educational environments (Chenyang, 2022; Passarelli and Kolb, 2023; Shan et al., 2021). This implies that culturally intelligent students become multiculturally literate not only because they understand cultural differences, but because they can communicate across them effectively and empathetically. Accordingly, cross-cultural communication skills are positioned as a complementary mediator—alongside cultural exposure—in the development of multicultural literacy.

Based on this theoretical rationale and previous empirical findings, the following hypotheses are proposed:

H3a: Cultural intelligence is positively associated with cross-cultural communication skills.
H3b: Cross-cultural communication skills are positively associated with multicultural literacy.
H3c: Cross-cultural communication skills mediate the relationship between cultural intelligence and multicultural literacy.

### Relationship between cultural exposure and cross-cultural communication skills

While cultural exposure and cross-cultural communication skills represent distinct developmental processes, prior scholarship suggests they are often dynamically related rather than strictly independent. Experiential learning theory emphasizes that meaningful intercultural contact provides opportunities for individuals to practice and refine communication strategies in authentic settings (Passarelli and Kolb, 2023). When students participate in intercultural events, study-abroad programs, or sustained interaction with peers from different cultural backgrounds, they are frequently challenged to decode unfamiliar verbal and nonverbal cues, negotiate meaning, and adjust their interactional styles accordingly (Presbitero, 2021; Deardorff, 2006). Such authentic intercultural engagement creates experiential “learning laboratories,” where theoretical cultural knowledge and attitudinal openness are transformed into practical communicative adaptability (Ramirez, 2023; Sharma and Rakhimova, 2024).

Interpersonal and intercultural competence models further indicate that effective communication behaviors are rarely developed in isolation; they are honed through iterative interaction with culturally diverse others (Chen, 2024; Hashimoto, 2023). Students who experience higher levels of cultural exposure typically report greater intercultural sensitivity and confidence in cross-cultural interactions (Wawrosz and Jurásek, 2021). This suggests that cultural exposure functions not only as a direct enabler of multicultural literacy but also as a precursor to the acquisition of communication skills that are critical for leveraging diversity in meaningful ways. Conversely, individuals lacking sufficient exposure may possess cultural awareness but lack the communicative agility required to enact that awareness in practice.

Drawing on these theoretical insights, it is plausible that cultural exposure and cross-cultural communication skills together form a developmental sequence through which cultural intelligence is actualized into multicultural literacy. This sequential view aligns with socio-cognitive models of intercultural competence, which posit that intercultural adaptation emerges from iterative cycles of contact and reflective communication (Kolb, 2015; Deardorff, 2006). Accordingly, cultural exposure can be expected to strengthen students' communication competencies, which then serve as a conduit for achieving higher levels of multicultural literacy.

Based on this rationale, the following additional hypotheses are proposed:

H4a: Cultural exposure is positively associated with cross-cultural communication skills.
H4b: Cultural exposure and cross-cultural communication skills sequentially mediate the relationship between cultural intelligence and multicultural literacy.

### Theoretical framework

This study draws on theoretical perspectives from cultural intelligence theory (Rachmad, 2022), experiential learning, and intercultural communication competence to conceptualize how multicultural literacy develops in university students. Cultural intelligence is defined as an individual's capability to function effectively in culturally diverse settings and is considered a multidimensional construct encompassing cognitive, motivational, and behavioral components (Malay et al., 2022). As a meta-competence, cultural intelligence provides the foundational awareness, sensitivity, and adaptability that enable individuals to interpret and respond to cultural differences. However, scholars increasingly recognize that cultural intelligence alone does not automatically translate into intercultural competence or multicultural literacy without supportive contexts and enabling behavioral mechanisms (Shliakhovchuk, 2021). Therefore, investigating the processes through which cultural intelligence contributes to multicultural development is essential.

From an experiential learning perspective, cultural exposure serves as a situational enabler that provides real-life contexts in which individuals can apply, challenge, and expand their cultural knowledge (Ramirez, 2023). Exposure to diverse cultural settings—whether through friendships, study programs, or digital content—facilitates contact-based learning, promotes reflection, and triggers the activation of cultural intelligence components in practice. Parallel to this, cross-cultural communication skills are rooted in interpersonal and intercultural competence theories, emphasizing the ability to encode, decode, and negotiate meaning across cultural boundaries (Sharma and Rakhimova, 2024). These skills reflect one's capacity to translate internal awareness into external interaction and are influenced by underlying traits such as empathy, openness, and cultural sensitivity. Both mediators reflect distinct dimensions of the developmental process: cultural exposure represents opportunity structures for learning, while cross-cultural communication reflects the capacity to interact effectively within those opportunities.

Importantly, experiential and intercultural competence models suggest that these two processes are often mutually reinforcing. Authentic exposure to culturally diverse contexts provides opportunities to practice and refine communicative flexibility, while enhanced communication skills enable individuals to engage more deeply and effectively in subsequent intercultural interactions (Passarelli and Kolb, 2023; Presbitero, 2021; Deardorff, 2006). This developmental interplay implies that cultural exposure may contribute not only directly to multicultural literacy but also indirectly by fostering cross-cultural communication skills, which then translate awareness into effective intercultural engagement. Incorporating both independent and sequential pathways provides a more holistic account of how psychological and experiential factors jointly shape multicultural literacy.

This study focuses on Chinese university students because China's higher education system has undergone rapid internationalization in recent years, resulting in increased multicultural interactions on campuses (Chen, 2025; Liu and Lim, 2024). However, despite this shift, empirical research examining how Chinese students develop multicultural literacy through both experiential and communicative pathways remains limited. Understanding these dynamics in the Chinese context is critical, as the country's unique socio-cultural environment, educational norms, and language dynamics may shape the way cultural intelligence translates into multicultural competencies.

Multicultural literacy is conceptualized as the ability to understand, respect, and navigate multiple cultural perspectives, but it also entails critical awareness of cultural assumptions, the capacity to mediate between differing worldviews, and the skill to apply such knowledge in contextually appropriate ways (Ahadiyyah et al., 2024; Qiu et al., 2024). While related to constructs such as intercultural competence and global mindset, multicultural literacy places greater emphasis on the synthesis of cognitive, affective, and behavioral capacities into an integrated, context-sensitive form of cultural understanding (Yan, 2025). Intercultural competence often focuses on performance in cross-cultural interactions, and global mindset emphasizes openness to global opportunities; in contrast, multicultural literacy foregrounds the interpretive and reflective skills that allow individuals to critically engage with diversity across both local and global contexts (Wu, 2025). This distinction positions multicultural literacy as both an outcome of and a prerequisite for sustained intercultural engagement.

The proposed framework positions cultural intelligence as the central antecedent, while cultural exposure and communication skills function as complementary and developmentally linked mediators that channel cultural intelligence into literacy outcomes. By recognizing that exposure can enhance communication competencies, which then further support multicultural literacy, the framework captures both the independent and sequential contributions of these processes. This integrative model aligns with socio-cognitive models of intercultural development, which argue that cultural competence emerges not only from internal traits but also through repeated interaction and behavioral engagement with diversity (Zhou and Colomer, 2024). By empirically testing these interrelationships, the study aims to provide a theoretically grounded and practically relevant explanation of how university students in multicultural learning environments develop literacy across cultural contexts.

**Figure 1:**
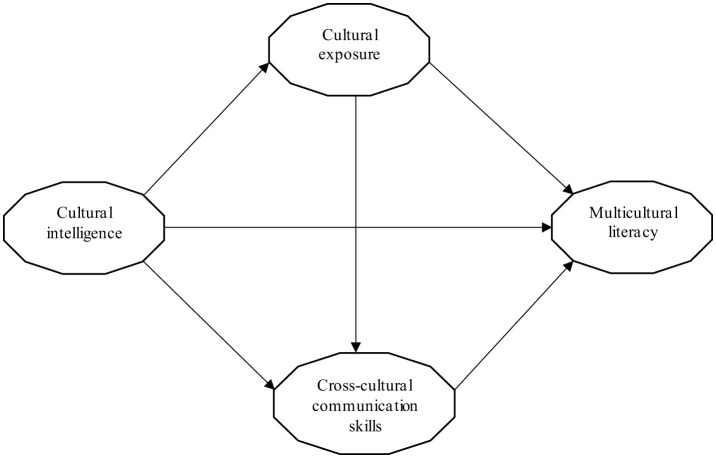
Conceptual model.

The theoretical framework is depicted in Figure 1 below:

## Materials and method

### Participants

This study targeted university students enrolled in undergraduate programs across multiple provinces in mainland China. University students were selected because they are at a developmental stage where multicultural learning, identity negotiation, and intercultural engagement are most salient, as noted by Deardorff (2006) and Zhou et al. (2008). Moreover, their frequent exposure to global influences through coursework, social media, and peer interactions makes them particularly suitable for examining variables such as cultural intelligence, cultural exposure, cross-cultural communication skills, and multicultural literacy. In addition, Chinese university students were recruited because China's higher education sector is undergoing rapid internationalization, marked by increasing numbers of inbound and outbound student exchanges, expansion of English-medium programs, and diversification of campus populations (Qiu et al., 2024; Shadiev et al., 2021). In this context, multicultural exposure typically occurs through observable opportunities such as interaction with international students enrolled in joint-degree or exchange programs, participation in English-taught courses, involvement in cross-cultural student clubs, and attendance at campus events like international culture festivals and language exchange activities. Even where demographic diversity is relatively limited, these structured and informal initiatives create meaningful avenues for intercultural contact and reflective learning. These shifts create a dynamic environment where intercultural engagement is both a strategic priority and a practical challenge. Studying this issue in the Chinese context offers valuable insights into how multicultural literacy develops in a large, non-Western higher education system that is actively shaping global academic mobility.

To ensure proportional representation across academic backgrounds, a stratified random sampling method was employed. This technique was used to include students from diverse disciplines such as business, education, international studies, and social sciences, which allowed for broader generalizability and minimized sampling bias, consistent with recommendations by Thompson (2012). The sample was drawn from four comprehensive universities located in three provinces (Jiangsu, Guangdong, and Sichuan), representing both Project 211 universities and regional teaching-focused institutions to capture variation in institutional tier and degree of internationalization. Within each academic stratum, participants were randomly selected using enrollment records and classroom lists. Data were collected in person by trained research assistants who provided participants with a brief overview of the study, obtained informed consent, and distributed the paper-based questionnaires. Participants were assured that their responses would remain confidential and anonymous, and no identifying information was collected. All ethical procedures were approved by the institutional review board of the affiliated institution, in accordance with the Declaration of Helsinki. A total of 500 questionnaires were distributed over a 12-week data collection period. Of these, 419 were returned, yielding a response rate of 83.8 percent. After data screening, which included the removal of incomplete, patterned, or inconsistent responses, 412 valid responses were retained for analysis.

### Research instruments

#### Cultural intelligence

Cultural intelligence was assessed using a 10-item short form adapted from the Cultural Intelligence Scale developed by Thomas et al. (2015). This scale measures individuals' capability to function effectively across various cultural contexts. Sample items include “I adjust my communication appropriately when with culturally diverse individuals” and “I reflect on how cultural backgrounds shape behavior.” Responses were rated on a 5-point Likert scale ranging from 1 (strongly disagree) to 5 (strongly agree). Higher scores indicate greater levels of cultural intelligence. The scale has demonstrated satisfactory internal consistency in previous studies (Cronbach's alpha > 0.80).

#### Cultural exposure

Cultural exposure was measured using a 6-item scale adapted from intercultural learning and experience literature (e.g., Hammer, 2023; Sousa et al., 2023). The items capture students' engagement with culturally diverse environments and experiences. A sample item includes “I frequently participate in events involving people from other cultures.” Responses were rated on a 5-point Likert scale (1 = strongly disagree to 5 = strongly agree), with higher scores indicating greater cultural exposure.

#### Cross-cultural communication skills

Cross-cultural communication skills were measured using 7 items adapted from Cegala (1981) interaction involvement scale and extended by Chen and Starosta (2012). This scale assesses respondents' ability to communicate effectively across cultural boundaries, including clarity, adaptability, and responsiveness. Sample items include “I adjust my tone and vocabulary when communicating with people from different cultures” and “I seek feedback to check if my message was understood across cultures.” All items used a 5-point Likert scale (1 = strongly disagree to 5 = strongly agree).

#### Multicultural literacy

Multicultural literacy was assessed using a 6-item scale derived from multicultural personality and intercultural development frameworks (e.g., Bennett, 1993; Van der Zee and Van Oudenhoven, 2000). This scale measures the extent to which individuals recognize, respect, and integrate diverse cultural perspectives. Sample items include “I respect different cultural viewpoints, even when they conflict with mine” and “I consider multiple cultural perspectives when analyzing social or academic issues.” Responses were recorded on a 5-point Likert scale, with higher scores reflecting greater multicultural literacy.

### Statistical analysis

All statistical analyses were conducted using SPSS version 26 and SmartPLS version 4.1.0.6. Data analysis was performed using SmartPLS version 4.1.0.6 under an academic license. The PLS-SEM algorithm was run with a maximum of 300 iterations, a stop criterion of 10^−^7, and the path weighting scheme. Missing data were handled using listwise deletion, and bootstrapping was conducted with 5,000 subsamples under default settings.

Descriptive statistics, including means, standard deviations, and frequency distributions, were calculated to summarize the demographic characteristics and variable distributions. Prior to hypothesis testing, data screening procedures such as missing value analysis, normality checks, and outlier detection were performed. Structural Equation Modeling (SEM) with Partial Least Squares (PLS) was employed to examine the hypothesized serial mediation model. PLS-SEM was chosen due to its suitability for predictive modeling and its robustness in handling complex models with multiple mediators and relatively small to medium sample sizes (Hair et al., 2019; Sarstedt et al., 2022). The bootstrapping technique with 5,000 subsamples was used to test the significance of direct and indirect effects. Measurement model assessment involved evaluating construct reliability, convergent validity (using Average Variance Extracted), and discriminant validity (using Fornell–Larcker criterion and heterotrait-monotrait (HTMT) ratios). Model fit was assessed using SRMR and NFI indices, following recommended cut-offs for PLS-based models.

### Participants demographic profiles

The final sample consisted of 53.2 percent female (*n* = 219) and 46.8 percent male (*n* = 193) students. Participants ranged in age from 18 to 25 years, with a mean age of 20.6 years and a standard deviation of 1.7. In terms of academic discipline, 34.5 percent of the students were majoring in business, 22.8 percent in education, 18.7 percent in international studies, and 24.0 percent in social sciences. Regarding year of study, 25.2 percent were first-year students, 29.4 percent were in their second year, 23.3 percent were third-year students, and 22.1 percent were in their final year. Furthermore, 64.6 percent of the respondents reported having interacted with international students or having participated in intercultural events on campus (Table 1).

**Table 1:** Sample characteristics.

**Characteristic**	***n***	**%**
**Gender**		
Female	219	53.2
Male	193	46.8
**Age (years)**		
Mean (SD)		20.6 (1.7)
Range		18–25
**Academic discipline**		
Business	142	34.5
Education	94	22.8
International studies	77	18.7
Social sciences	99	24.0
**Year of study**		
First year	104	25.2
Second year	121	29.4
Third year	96	23.3
Final year	91	22.1
**Intercultural interaction**		
Yes	266	64.6

### Measurement model evaluation

#### Psychometric properties of constructs

The Table 2 presents the psychometric properties of the four constructs used in the study, including item loadings, Cronbach's alpha, composite reliability, and average variance extracted. For cross-cultural communication skills, the construct showed good internal consistency with a Cronbach's alpha of 0.858, composite reliability of 0.892, and average variance extracted of 0.543. Item loadings ranged from 0.655 to 0.798, suggesting acceptable convergent validity despite a few items loading slightly below the ideal 0.70 threshold. The construct cultural exposure demonstrated strong reliability, with Cronbach's alpha of 0.881, composite reliability of 0.913, and average variance extracted of 0.678. Item loadings varied from 0.777 to 0.847, with most items exceeding 0.70, except CE4, which was eliminated due to poor loading. Cultural intelligence yielded a Cronbach's alpha of 0.860, composite reliability of 0.887, and average variance extracted of 0.500, which meets the minimum criteria for convergent validity. Item loadings for this construct ranged from 0.692 to 0.779. Items indicating poor loadings < 0.60 were dropped from the subsequent analysis (Hair et al., 2019). For multicultural literacy, the construct demonstrated strong internal reliability, with a Cronbach's alpha of 0.842, composite reliability of 0.884, and average variance extracted of 0.560. Item loadings were all above 0.65, ranging from 0.658 to 0.804, supporting the construct's convergent validity and internal consistency. Collectively, these values confirm that the constructs used in this study exhibit acceptable levels of reliability and validity for further structural model analysis (Hair et al., 2019; Fornell and Larcker, 1981; Nunnally and Bernstein, 1994).

**Table 2:** Constructs psychometric properties.

**Items**	**Loadings**	**CA**	**CR**	**AVE**
Cross-cultural communication skills		0.858	0.892	0.543
CCCS1	0.691			
CCCS2	0.676			
CCCS3	0.778			
CCCS4	0.798			
CCCS5	0.773			
CCCS6	0.771			
CCCS7	0.655			
Cultural exposure		0.881	0.913	0.678
CE1	0.847			
CE2	0.846			
CE3	0.777			
CE5	0.817			
CE6	0.829			
Cultural intelligence		0.852	0.884	0.523
CI1	0.692			
CI10	0.779			
CI3	0.758			
CI4	0.725			
CI5	0.681			
CI6	0.762			
CI8	0.657			
Multicultural literacy		0.842	0.884	0.560
ML1	0.804			
ML2	0.795			
ML3	0.782			
ML4	0.737			
ML5	0.658			
ML6	0.703			

#### Heterotrait-monotrait ratio: discriminant validity

Table 3 reports the heterotrait–monotrait (HTMT) ratios with bootstrapped 95% confidence intervals for all construct pairs. All HTMT values remain below the conservative 0.90 benchmark typically recommended for conceptually related constructs (Henseler et al., 2015; Hair et al., 2019), confirming adequate discriminant validity. The strongest association is between multicultural literacy and cultural exposure (HTMT = 0.848; mean = 0.851), with the confidence interval ranging from 0.777 to 0.88 Although this reflects a close conceptual relationship, the value is well below the 0.90 threshold, and the upper bound remains far from 1.0, indicating that the constructs are empirically distinct. The cultural intelligence–cultural exposure link is moderate (0.715; CI upper bound 0.800), while all other construct pairs fall between 0.615 and 0.760 with upper bounds below 0.86. The narrow bootstrapped intervals further attest to the stability of these estimates. Taken together, the HTMT findings, along with the Fornell–Larcker results (presented in the subsequent section), provide strong evidence of discriminant validity across all study constructs.

**Table 3:** Heterotrait-monotrait (HTMT) ratio—confidence intervals.

**Constructs**	**Original sample (O)**	**Sample mean (M)**	**2.5%**	**97.5%**
CE < -> CCCS	0.760	0.762	0.662	0.859
CI < -> CCCS	0.615	0.610	0.509	0.708
CI < -> CE	0.715	0.713	0.630	0.800
ML < -> CCCS	0.745	0.746	0.650	0.826
ML < -> CE	0.848	0.851	0.777	0.889
ML < -> CI	0.680	0.681	0.583	0.770

#### Fornell-Larcker criterion: discriminant validity

Discriminant validity was examined using both the Fornell–Larcker criterion and the heterotrait–monotrait (HTMT) ratio with bootstrapped confidence intervals. As shown in Table 4, the square roots of AVE (diagonal elements) are greater than the corresponding inter-construct correlations in the rows and columns below, which satisfies the Fornell–Larcker criterion (Fornell and Larcker, 1981). For instance, the square root of AVE for ML is 0.748, which exceeds its correlations with cross-cultural communication skills (0.639), cultural exposure (0.709), and cultural intelligence (0.628). Similarly, cultural exposure's AVE square root (0.824) is higher than its correlations with cross-cultural communication skills (0.667) and cultural intelligence (0.694), confirming that each construct shares more variance with its own indicators than with other constructs.

**Table 4:** Fornell-Larcker criterion.

**Constructs**	**CCCS**	**CE**	**CI**	**ML**
CCCS	0.737			
CE	0.667	0.824		
CI	0.563	0.694	0.706	
ML	0.639	0.709	0.628	0.748

#### Variance inflation factor: collinearity statistics

Collinearity was assessed using inner-model variance inflation factors (VIF) for all predictor constructs (Table 5). All VIF values are well below the commonly recommended threshold of 5.0 (Hair et al., 2019), indicating that multicollinearity is not a concern in the structural model. The VIFs range from 1.000 for cultural intelligence predicting cultural exposure to 2.456 for cultural exposure predicting multicultural literacy. Cross-cultural communication skills and cultural intelligence also show acceptable VIFs when predicting multicultural literacy (1.866 and 1.998, respectively), and the VIF values for cultural exposure and cultural intelligence when predicting cross-cultural communication skills are both 1.928. These results confirm that the predictor variables do not exhibit problematic levels of collinearity and that the model's structural paths can be interpreted without bias due to multicollinearity (Kock, 2015).

**Table 5:** Collinearity statistics (VIF)—inner model—matrix.

**Constructs**	**CCCS**	**CE**	**CI**	**ML**
CCCS				1.866
CE	1.928			2.456
CI	1.928	1.000		1.998
ML				

Additionally, Harman's single-factor test was conducted to further examine common method bias. The results showed that the first unrotated factor accounted for less than 40% of the total variance, indicating that no single factor dominated and that common method bias is unlikely to threaten the validity of the findings (Appendix 1).

### Structural model results

#### Path coefficient

Table 6 presents the path coefficients with bias-corrected 95% bootstrapped confidence intervals. All hypothesized relationships are positive and significant, as the respective confidence intervals do not include zero. Cultural intelligence has a significant direct effect on multicultural literacy [β = 0.131, 95% CI (0.042, 0.222)]; therefore, Hypothesis 1 is supported. Cultural intelligence significantly predicts cultural exposure (β = 0.711, 95% CI (0.661, 0.756)]; hence, Hypothesis 2a is accepted. Cultural exposure has a significant positive effect on multicultural literacy [β = 0.557, 95% CI (0.440, 0.671)]; therefore, Hypothesis 2b is supported. Cultural intelligence significantly influences cross-cultural communication skills [β = 0.192, 95% CI (0.060, 0.313)]; thus, Hypothesis 3a is accepted. Cross-cultural communication skills significantly predict multicultural literacy (β = 0.194, 95% CI [0.083, 0.310]); therefore, Hypothesis 3b is supported. Cultural exposure significantly predicts cross-cultural communication skills [β = 0.530, 95% CI (0.388, 0.669)]; hence, Hypothesis 4a is accepted. These findings collectively validate all hypothesized relationships proposed in the conceptual model.

**Table 6:** Path coefficients—confidence intervals bias-corrected.

**Relationships**	**Original sample (O)**	**Sample mean (M)**	**Bias**	**2.5%**	**97.5%**
CI -> ML	0.131	0.138	0.007	0.042	0.222
CI -> CE	0.711	0.712	0.001	0.661	0.756
CI -> CCCS	0.192	0.185	−0.007	0.060	0.313
CE -> ML	0.557	0.558	−0.002	0.440	0.671
CCCS -> ML	0.194	0.191	−0.001	0.083	0.310
CE -> CCCS	0.530	0.539	0.007	0.388	0.669

#### Effect size: f-square

Effect sizes (f^2^) were examined to assess the contribution of each exogenous construct to its respective endogenous constructs (Table 5). According to Cohen (1988) guidelines, f^2^ values of 0.02, 0.15, and 0.35 can be interpreted as small, medium, and large effects, respectively. The analysis indicates that CI has a very large effect on CE (f^2^ = 0.928), which underscores the strong predictive relevance of cultural intelligence for cultural exposure. This high value reflects substantive explanatory power and is not indicative of multicollinearity, as supported by the acceptable HTMT and Fornell–Larcker results reported earlier. CI also shows small effects on CCCS (f^2^ = 0.036) and ML (f^2^ = 0.024), while CE demonstrates large effects on ML (f^2^ = 0.357) and medium effects on CCCS (f^2^ = 0.274). CCCS exhibits a small effect on ML (f^2^ = 0.055) (Table 7).

**Table 7:** f-square—matrix.

**Constructs**	**CCCS**	**CE**	**CI**	**ML**
CCCS				0.055
CE	0.274			0.357
CI	0.036	0.928		0.024
ML				

Table 8 presents the bias-corrected bootstrapped confidence intervals for the specific indirect effects. The indirect effect of cultural intelligence on multicultural literacy through cultural exposure is positive and significant [β = 0.396, 95% CI (0.3, 0.475)], indicating support for the mediating role of cultural exposure as hypothesized in Hypothesis 2c. The indirect effect of cultural intelligence on multicultural literacy through cross-cultural communication skills is also positive and significant [β = 0.037, 95% CI (0.012, 0.080)], providing support for Hypothesis 3c. Finally, the serial mediation pathway from cultural intelligence to cultural exposure, from cultural exposure to cross-cultural communication skills, and from cross-cultural communication skills to multicultural literacy is positive and significant [β = 0.073, 95% CI (0.028, 0.134)], confirming Hypothesis 4b. These findings demonstrate that cultural exposure serves as the primary mediator, while cross-cultural communication skills also contribute both independently and sequentially, reinforcing the theoretical rationale for multiple mediation mechanisms proposed in this study.

**Table 8:** Specific indirect effects—confidence intervals bias-corrected.

**Relationships**	**Original sample (O)**	**Sample mean (M)**	**Bias**	**2.5%**	**97.5%**
CI -> CE -> ML	0.389	0.388	−0.001	0.305	0.466
CI -> CCCS -> ML	0.037	0.035	−0.003	0.012	0.080
CI -> CE -> CCCS -> ML	0.071	0.072	0.00	0.028	0.134

**Figure 2:**
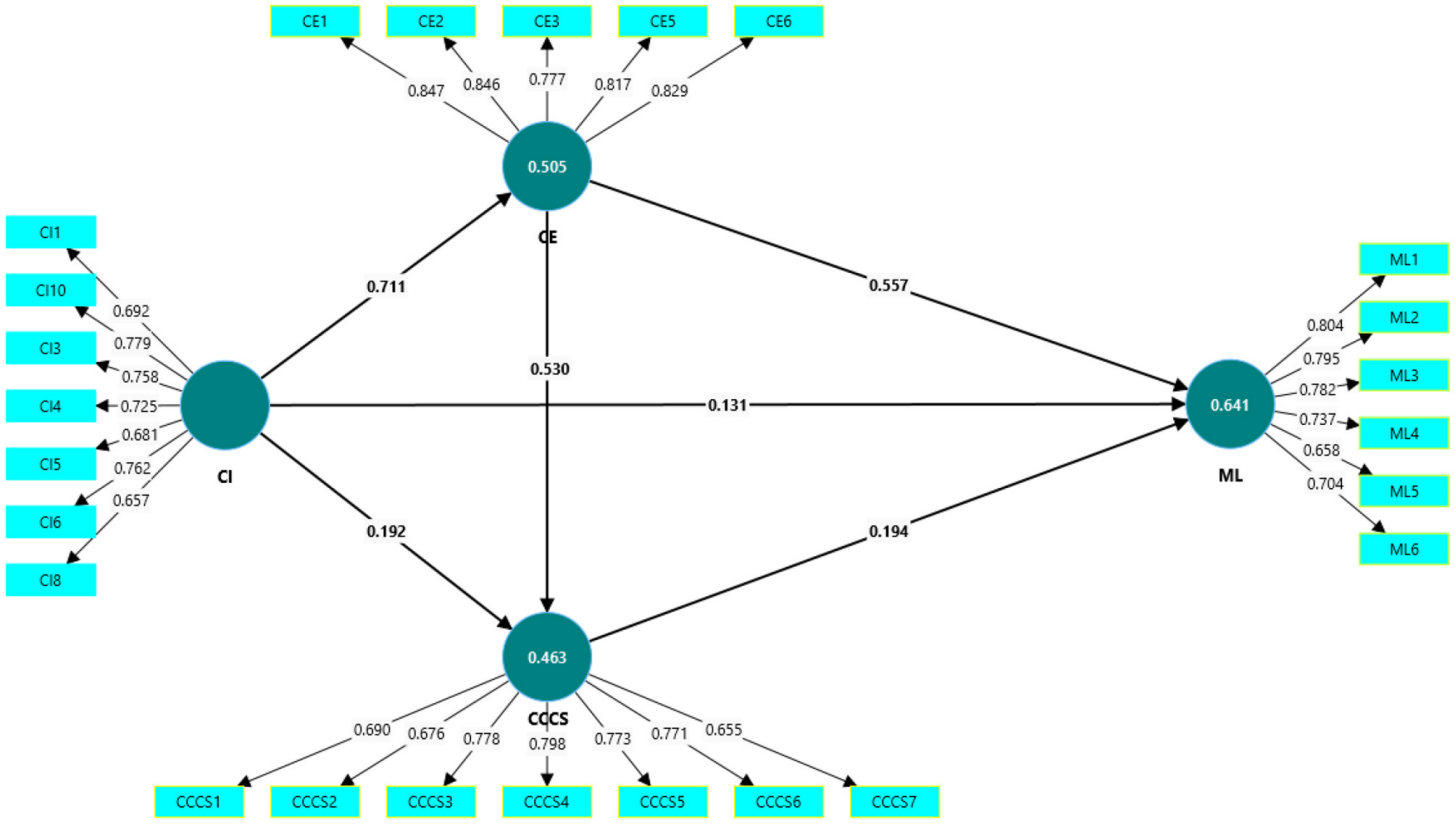
PLS algorithm output.

The PLS algorithm output presented in Figure 2 illustrates the structural relationships among the study variables. In this model, all hypothesized paths are presented with standardized path coefficients, which indicate both the strength and the direction of each relationship. Cultural intelligence demonstrated strong predictive relationships with both cultural exposure and cross-cultural communication skills, while cultural exposure and cross-cultural communication skills in turn showed significant positive effects on multicultural literacy. The figure also reports the R^2^ values for the endogenous constructs, highlighting that the model explains a substantial proportion of the variance in multicultural literacy (R^2^ = 0.641), as well as notable variance in cultural exposure (R^2^ = 0.505) and cross-cultural communication skills (R^2^ = 0.463). This demonstrates the overall explanatory power of the proposed framework.

#### Model fit

Model fit was assessed using several standard indices. The standardized root mean square residual (SRMR) was 0.046, which is below the conservative cut-off of 0.08 recommended for PLS-SEM (Henseler et al., 2015). The normed fit index (NFI) was 0.91, exceeding the commonly accepted threshold of 0.90, indicating good model fit (Hu and Bentler, 1999). Additionally, the chi-square statistic (χ^2^) associated with NFI was 1,230.45 (df = 645), yielding a χ^2^/df ratio of 1.91, which falls below the acceptable benchmark of 3.0 (Hair et al., 2019). These results collectively support the adequacy of the model fit.

## Discussion

The findings of this study offer several meaningful contributions to theory within the domains of intercultural competence, educational psychology, and social learning. First, the study advances cultural intelligence theory (Rachmad, 2022) by demonstrating that its influence on multicultural literacy is not solely direct but also unfolds through multiple mediating mechanisms. While previous research has established cultural intelligence as a predictor of intercultural effectiveness (Figueroa and Hofhuis, 2024; Schelfhout et al., 2022; Skaria and Montayre, 2023), the present findings show that its impact depends substantially on students' experiential and behavioral engagement with diversity. This aligns with recent scholarship emphasizing that the benefits of cultural intelligence are maximized when individuals are provided with authentic opportunities to apply their intercultural capabilities (Qiu et al., 2024; Tripathi et al., 2024). Rather than viewing cultural intelligence as a static trait, these results underscore its role as an enabling resource that becomes meaningful through lived experiences and enacted behaviors.

Second, by incorporating experiential learning theory (Nelson and Luetz, 2021; Passarelli and Kolb, 2023), this study highlights the role of cultural exposure as an active learning condition for multicultural development. Prior models of multicultural learning often focus on curriculum design or classroom pedagogy; however, our results indicate that engagement with cultural diversity outside formal instruction—through informal interactions, campus events, and intercultural activities—serves as a critical bridge between cultural awareness and deeper literacy (Smith and Warrican, 2021). This is consistent with higher education research suggesting that sustained exposure to diverse perspectives fosters critical cultural reflection and global-mindedness beyond the classroom (Jiang et al., 2024; Zhang, 2024). Importantly, our serial mediation findings reveal that cultural exposure not only influences multicultural literacy directly but also enhances students' cross-cultural communication skills, which in turn further strengthens multicultural literacy. This developmental sequence supports socio-cognitive models positing that experiential opportunities provide the foundation upon which communicative adaptability can be honed over time.

Third, the inclusion of cross-cultural communication competence as both an independent and sequential mediator underscores the behavioral dimension of multicultural development. Intercultural learning frameworks frequently emphasize knowledge and attitudes, but our findings show that communicative adaptability and skillful interaction are equally critical to literacy outcomes. In the Chinese higher education context, the cultural exposure pathway emerged as comparatively stronger than the direct behavioral pathway. This pattern can be explained by the demographic homogeneity of Mainland Chinese universities, where opportunities for deep intercultural dialogue are often limited. Under such conditions, even modest or structured forms of exposure—such as participation in international festivals, short-term interaction with exchange students, English-medium instruction, and online collaborative projects—can have an immediate and salient impact on multicultural literacy. These “quasi–cross-cultural” experiences provide powerful experiential input despite limited demographic diversity, whereas the benefits of communication skills likely accumulate gradually through repeated practice and reflection. This mechanism-based account helps explain why cultural exposure serves as a primary driver of literacy in this context, consistent with prior work showing that experiential opportunities often precede and enable the full benefits of communication competence (Presbitero, 2021; Jurásek and Wawrosz, 2024). These findings reinforce calls for integrative models that combine affective, cognitive, and behavioral components of intercultural competence (Alhendi, 2021), while also validating the inclusion of communication skills as an outcome-relevant construct in multicultural education.

Finally, by testing both independent and serial mediation pathways, the study contributes a more nuanced theoretical understanding of how multiple mechanisms jointly explain the development of multicultural literacy. The results challenge overly linear models by showing that environmental engagement (via cultural exposure) and behavioral enactment (via communication skills) operate in sequence as well as independently, offering a multidimensional perspective on how cultural intelligence translates into multicultural literacy. This advances prior research on intercultural competence by empirically demonstrating that experiential and behavioral pathways can coexist and reinforce each other. At the same time, these findings should be interpreted with appropriate bounds of generalization. As the study focused on undergraduates in Mainland China, where intercultural contact is relatively constrained, the results are most applicable to educational environments characterized by low baseline diversity but increasing internationalization. Future studies should test whether the observed strength of the cultural exposure pathway holds in more demographically diverse settings or among populations with greater intercultural experience to delineate boundary conditions and enhance the generalizability of these findings (Steyn and Vanyoro, 2024; Ahadiyyah et al., 2024). Overall, the multidimensional mediation model offered here provides a richer theoretical lens for understanding student development in increasingly globalized yet contextually varied higher education systems.

## Practical implications

The findings of this study offer valuable insights for educators, university administrators, and policymakers seeking to cultivate multicultural literacy among students in increasingly diverse and globalized educational environments. First, the significant role of cultural intelligence suggests the need to embed intercultural competence training into higher education curricula. Universities can implement targeted interventions such as cultural intelligence workshops, reflective modules, and case-based simulations to help students develop cognitive flexibility, empathy, and cross-cultural awareness. These modules could be designed as semester-long units embedded in general education courses, incorporating activities such as cultural self-assessments, scenario-based role-plays, and problem-solving simulations involving diverse cultural perspectives. These activities can serve as foundational tools for preparing students to navigate complex cultural dynamics both within and beyond the academic context.

Second, given the comparatively stronger contribution of cultural exposure in our model, institutions should prioritize creating and sustaining genuine and sustained opportunities for students to engage with cultural diversity. Strategies such as study-abroad programs, international student mentorship schemes, and multicultural campus events should be emphasized as central initiatives. For example, universities could offer short-term international field visits aligned with course objectives, host annual multicultural festivals co-led by domestic and international student organizations, and implement “culture labs” where students collaboratively document and present intercultural experiences. Even in domestic settings, universities can design local intercultural engagement projects—such as language exchange partnerships or community-based learning with migrant groups—that foster real-life exposure to diverse perspectives. By emphasizing consistent and authentic exposure opportunities, institutions can maximize the primary mechanism identified in this study that drives multicultural literacy.

Third, while cultural exposure should take precedence, the influence of cross-cultural communication skills remains important as a complementary pathway. Course content and co-curricular programs should include activities that promote interpersonal adaptability, intercultural dialogue, and collaborative problem-solving. Examples include structured intercultural debate series, virtual exchange programs connecting students with peers abroad for joint projects, and peer-mentoring systems where students from different linguistic and cultural backgrounds co-develop presentations or research reports. Group assignments that require students from different cultural backgrounds to work together can provide a structured environment for developing communication skills while enhancing mutual understanding. Furthermore, training programs for academic staff can equip instructors with the skills necessary to model and facilitate inclusive communication practices in diverse classrooms.

Together, these implications suggest that advancing multicultural literacy requires a multipronged strategy that prioritizes authentic and sustained cultural exposure while supplementing it with communication skills training and psychological readiness programs. By focusing institutional efforts on experiential diversity as the primary driver, and supporting it with interpersonal skill-building, higher education institutions can more effectively prepare students to thrive in multicultural and globally interconnected societies.

## Limitations and directions for future studies

While this study offers important insights into the mechanisms linking cultural intelligence and multicultural literacy, several limitations should be acknowledged. First, the cross-sectional design restricts causal interpretation of the proposed relationships. Longitudinal or experimental studies are recommended to confirm the directionality of effects and assess changes over time. Second, the data were collected solely from university students in China, which may limit the generalizability of findings to other cultural or educational contexts. Future research should test the model across diverse populations to enhance external validity. The cultural, institutional, and policy-specific characteristics of Chinese higher education may shape the relationships observed in ways that differ from other regions. To strengthen external validity, future research should replicate this model in varied contexts—such as universities in Western, African, and other Asian countries—and compare results across cultural settings to identify both universal and context-specific patterns.

Third, reliance on self-reported measures introduces the potential for common method bias and social desirability effects. Incorporating peer ratings, behavioral observations, or mixed-method designs in future studies would help mitigate these concerns. Lastly, the model focused on two mediators; however, additional variables such as empathy, intercultural sensitivity, or institutional diversity climate may also influence multicultural literacy. Future work could expand the framework by exploring moderated mediation models to capture these complex dynamics.

## Conclusion

In an era of increasing global interconnectedness, fostering multicultural literacy among university students has become a critical educational priority. This study contributes to the growing literature by demonstrating that cultural intelligence significantly predicts multicultural literacy both directly and indirectly through cultural exposure and cross-cultural communication skills. The findings highlight that psychological readiness alone is insufficient without opportunities for intercultural engagement and the behavioral capacity to navigate cultural interactions effectively. By integrating cognitive, experiential, and communicative dimensions into a unified framework, the study offers a nuanced understanding of how multicultural competencies are developed in higher education. These insights provide both theoretical clarity and practical direction for institutions aiming to equip students with the skills necessary to thrive in diverse cultural environments.

## Data Availability

The original contributions presented in the study are included in the article/Supplementary material, further inquiries can be directed to the corresponding author.

## Appendix

**Appendix 1:** Harman's single factor test

**Factor**	**Eigenvalue**	**% of variance explained (unrotated)**	**Cumulative %**
1	10.52	38.7%	38.7%
2	3.96	14.6%	53.3%
3	2.75	10.1%	63.4%
4	1.85	6.8%	70.2%
5+	< 1.00	Remaining factors	100%
