# Supplementary material for: Exploring the impact of cultural intelligence on multicultural literacy in university students: a serial mediation model of cultural exposure and cross-cultural communication skills
Source: Front Psychol. 2025 Sep 19;16:1661899. doi: 10.3389/fpsyg.2025.1661899 (PMC12491311; doi:10.3389/fpsyg.2025.1661899)
Supplement: Supplementary file 1 [file Supplementary_file_1.docx]

**Appendix I: Measurement scale items**

**Cultural intelligence**

1. I know the ways in which cultures around the world are different.
2. I can give examples of cultural differences from my personal experience.
3. I enjoy talking with people from different cultures.
4. I am confident I can adapt when interacting with people from different cultural backgrounds.
5. I am aware of different cultural norms in various settings.
6. I adjust my communication appropriately when with culturally diverse individuals.
7. I reflect on how cultural backgrounds shape behavior.
8. I enjoy learning unfamiliar cultural customs from others.
9. I can quickly understand cultural cues during interactions.
10. I adapt my behavior appropriately in diverse cultural contexts.

**Cultural exposure**

1. I frequently participate in events involving people from other cultures.
2. I have close friends from different cultural or ethnic backgrounds.
3. I actively seek opportunities to learn about cultures different from my own.
4. I have travelled to or lived in places with different cultural traditions.
5. I regularly consume international media (e.g., books, movies, or news from other cultures).
6. I engage with cultural festivals, exhibitions, or workshops outside my own culture.

**Cross-cultural communication skills**

1. I can clearly express my ideas when speaking with people from other cultures.
2. I actively listen and ask questions to ensure understanding in cross-cultural conversations.
3. I adjust my tone and vocabulary when communicating with people from different cultures.
4. I rephrase or explain my point if I feel I am not understood by someone from another culture.
5. I remain calm and respectful when communication misunderstandings occur.
6. I seek feedback to check if my message was understood across cultures.
7. I understand non-verbal cues in intercultural communication settings.

**Multicultural literacy**

1. I understand that people from different cultures may interpret situations differently.
2. I can recognize how my own cultural background influences how I see things.
3. I respect different cultural viewpoints, even when they conflict with mine.
4. I feel confident in situations involving people from various cultural backgrounds.
5. I consider multiple cultural perspectives when analyzing social or academic issues.
6. I can recognize and correct intercultural misunderstandings when they occur.
